# Orientation Selectivity in Inhibition-Dominated Networks of Spiking Neurons: Effect of Single Neuron Properties and Network Dynamics

**DOI:** 10.1371/journal.pcbi.1004045

**Published:** 2015-01-08

**Authors:** Sadra Sadeh, Stefan Rotter

**Affiliations:** Bernstein Center Freiberg & Faculty of Biology, University of Freiberg, Freiberg, Germany; Université Paris Descartes, Centre National de la Recherche Scientifique, France

## Abstract

The neuronal mechanisms underlying the emergence of orientation selectivity in the primary visual cortex of mammals are still elusive. In rodents, visual neurons show highly selective responses to oriented stimuli, but neighboring neurons do not necessarily have similar preferences. Instead of a smooth map, one observes a salt-and-pepper organization of orientation selectivity. Modeling studies have recently confirmed that balanced random networks are indeed capable of amplifying weakly tuned inputs and generating highly selective output responses, even in absence of feature-selective recurrent connectivity. Here we seek to elucidate the neuronal mechanisms underlying this phenomenon by resorting to networks of integrate-and-fire neurons, which are amenable to analytic treatment. Specifically, in networks of perfect integrate-and-fire neurons, we observe that highly selective and contrast invariant output responses emerge, very similar to networks of leaky integrate-and-fire neurons. We then demonstrate that a theory based on mean firing rates and the detailed network topology predicts the output responses, and explains the mechanisms underlying the suppression of the common-mode, amplification of modulation, and contrast invariance. Increasing inhibition dominance in our networks makes the rectifying nonlinearity more prominent, which in turn adds some distortions to the otherwise essentially linear prediction. An extension of the linear theory can account for all the distortions, enabling us to compute the exact shape of every individual tuning curve in our networks. We show that this simple form of nonlinearity adds two important properties to orientation selectivity in the network, namely sharpening of tuning curves and extra suppression of the modulation. The theory can be further extended to account for the nonlinearity of the leaky model by replacing the rectifier by the appropriate smooth input-output transfer function. These results are robust and do not depend on the state of network dynamics, and hold equally well for mean-driven and fluctuation-driven regimes of activity.

## Author Summary

It is not yet fully clear how sensory information is being processed when it arrives in primary cortical areas. We studied this general question in the context of rodent vision. We focused on the example of orientation selectivity, namely the selectivity of cortical neurons for specific orientations of an elongated stimulus. Our results show that a large body of experimental findings regarding the basic computations performed in early sensory processing can already be explained by linear processing of firing rates in neuronal networks with realistic parameters. The distribution of selectivity in our networks, as well as the exact shape of output tuning curves, including all details and inhomogeneities of structure and function, can be computed from the known connectivity matrix of the network and the known gain function of single neurons. A simple but essential form of nonlinearity, namely rectification of firing rates, accounts for sharpening of tuning curves and leads to some normalization of output modulation, even in networks without feature-specific connectivity. Our results and analyses demonstrate that none of these functional properties depend crucially on a fluctuation-driven or a mean-driven regime of activity, and that synchronous and asynchronous states of network dynamics can equally well serve these functions.

## Introduction

Orientation selectivity (OS), namely the selectivity of neurons in primary visual cortex (V1) of mammals to oriented stimuli [1]–[5], is the result of a simple computation in an early sensory cortex. However, thanks to its experimental and theoretical tractability, it provides a unique window to study the mechanisms underlying cortical computations [6]. The question of which mechanisms contribute to the emergence of OS and its contrast invariance (CI) [3], [7], [8] was recently revisited in light of new findings in the rodent visual cortex.

Although lacking smooth orientation maps on the cortical surface [9], highly selective receptive fields and sharp OS responses have been reported in these species [3]. It is still possible, though, that an invisible functional map of OS exists [10], as a result of the abundance of connections between neurons with similar OS [11]–[15], which may amplify OS responses [16]–[18]. Alternatively, other mechanisms can be at work already in networks without feature-specific connections, similar to the state of rodent visual cortex at the onset of eye opening [14].

It has indeed been shown that contrast invariant OS can emerge in balanced networks without feature-specific connections [19]–[21], and we asked here which mechanisms are essential to this process. Of special interest is what mechanisms on the level of cells or networks are responsible for selective attenuation of the baseline, and for selective amplification of the modulation in the input. Selective attenuation of the baseline ensures that the untuned component of the input is effectively suppressed, across different contrasts. As a result, the modulated component of the input contributes to reliable output spiking activity, without broadening the output tuning curves. Independent processing of these two signal components, baseline and modulation, ensures that the neurons respond selectively and reliably.

We investigate these mechanisms by studying large scale networks of perfect integrate-and-fire (PIF) and leaky integrate-and-fire (LIF) neurons. Our results show that comparable output OS, with sharp and CI responses, are obtained in both types of networks. The absence of a membrane leak in PIF networks, however, allows us to develop a fully linear, rate-based description of the network operation. Its analysis accounts for the main properties of OS – attenuation of the common mode, amplification of modulation, and contrast invariance – and explains their underlying neuronal mechanisms.

An inherent nonlinearity in spiking networks, namely rectification of negative “firing rates”, adds a distortion to the linear theory, and leads to new properties like sharpening of tuning curves and normalization. We further show that it is possible to account for this nonlinearity by extending the linear analysis in a straightforward way. In fact, by explicitly computing the exact shape of individual tuning curves, as well as the distribution of OS in our networks, and comparing them to the same quantities extracted from large-scale network simulations, we can safely exclude major contributions of other effects.

We demonstrate here that a large array of functional properties reported for biological sensory networks can already arise in networks without feature-specific connectivity, out of purely linear processes in the network, accompanied by the nonlinear process of rectification. The dynamic state of the network, synchronous or asynchronous, does not change our result, and all conclusions equally hold for both mean-driven and fluctuation-driven regimes of activity.

## Results

### Population tuning curves in recurrent networks

#### Network responses in the linear regime

**Figure 1:**
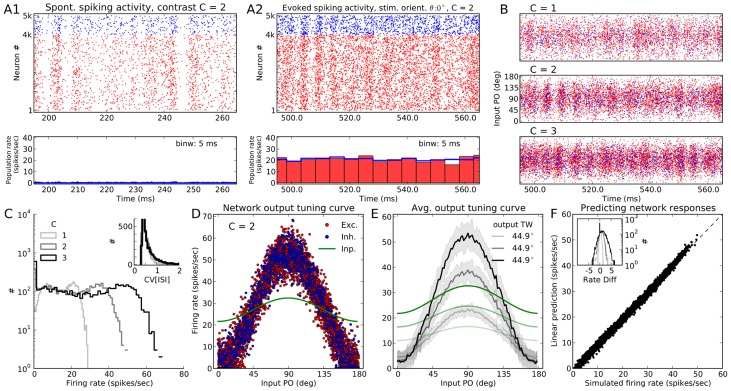
Neuronal responses in a network of PIF neurons to an oriented stimulus. (**A**) Spontaneous activity (A1) and evoked response of a PIF network to a stimulus of orientation 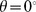 (A2) at a medium contrast, 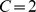. Evoked activity is the response of the network to the full external input (comprising the stimulus-independent background and the stimulus-induced feedforward input), whereas spontaneous activity of the network is the response to the background input only (for details, see Methods, Sect. *Simulation of networks of spiking neurons* and Eq. (1)). The network was arranged to have relatively weak recurrent inhibition, 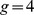. The spiking activity of the full network is shown for 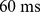 out of the full 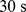 of stimulation. At the bottom, temporal population rates, computed in bins of size 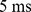, are shown for excitatory and inhibitory populations separately. Here and in the remaining text, unless otherwise stated, the colors red and blue are used to represent excitatory and inhibitory neurons, respectively. (**B**) Similar raster plot as in (A), top, for three different stimulus contrasts. However, neurons are now sorted according to the preferred orientation of their input (input PO). (**C**) Distribution of average firing rates estimated over 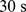, for different contrasts. Inset: Coefficient of variation (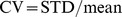) is computed from inter-spike intervals (ISI) of all neurons that emitted more than 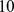 spikes during 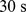 of stimulation at each contrast, respectively. (**D**) Average firing rate of neurons are plotted vs. their input PO, at the medium contrast (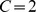). The feedforward (stimulus-induced) input to the network is normalized to the mean (over neurons) firing rate of the network and plotted in green for comparison. Exc.: Excitatory, Inh.: Inhibitory, Inp.: Input. (**E**) The mean (solid line) and STD (shading) of the population tuning curves, at each contrast. The range of input POs is broken into 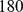 bins, and mean and STD are then computed from all neurons falling into the same bin. The average output tuning width (TW) at each contrast is computed from the von Mises function best-fitting to the mean output tuning curve (see Methods for details). The input to the network has a cosine tuning (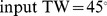), so any output TW less than this value implies a sharpening of the network tuning curves. The normalized feedforward input at each contrast is also plotted in green, similar to (D). (**F**) For each neuron in the network, prediction of the linear theory (Eq. (14)), is plotted vs. the firing rate obtained from the simulation at the medium contrast. The distributions of the difference between the actual and the predicted firing rates (rate diff) at this orientation are shown in the inset for all three contrasts, respectively.

We simulate the spiking activity of a network in response to 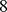 different stimulus orientations, one applied after the other, for 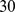 seconds each. The spiking activity of all neurons in the network in response to a stimulus with orientation 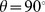 at an intermediate contrast (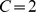) is shown in Fig. 1A. The network operates in the asynchronous-irregular (AI) state, as is evident from the raster plot and the peri-stimulus time histogram (PSTH) of the population activity. For the network simulated here, inhibitory synapses are arranged to be four times stronger than excitatory ones (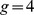); with the parameters used here, the network avoids rectification altogether (no “floor effect”). We have randomly drawn all the synaptic delays for recurrent connections (a uniform distribution on the interval 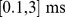), a choice that puts the network in the AI state.

If we sort the neurons according to their input POs (Fig. 1B), the selectivity of neurons becomes visible: Neurons with input POs far from the stimulus orientation respond with very low firing rates, and this is consistent across different contrasts. This is also evident from the distribution of firing rates across all neurons in the network (Fig. 1C), which has a very prominent peak at zero for all contrasts. The degree of irregularity of spike trains, quantified by the coefficient of variation (CV) of inter-spike intervals (ISI), also remains fairly stable upon stimulation with different contrasts (Fig. 1C, inset).

To better see the tuning of output responses, we can look at the tuning curves of all neurons by plotting the mean firing rate of neurons vs. the stimulus orientation relative to the input PO (Fig. 1D). The linear input-output response of the neuron model is visible here. Plotting the mean and standard deviation of the all tuning curves (Fig. 1E) reveals a robust output tuning for different contrasts. The large increase in the baseline component for higher contrasts does not carry over to the output tuning curves. The stimulus specific component, however, is amplified for all contrasts, for a range with a three-fold increase in the baseline intensity of the input. Average tuning curves of all neurons in the network, therefore, show high selectivity and contrast invariance.

#### Linear prediction of network responses

The mean output tuning curve of the network can be well predicted by a straightforward linear theory. For this stimulus orientation (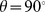), the prediction of Eq. 14 in the Methods is plotted vs. the result of simulation (Fig. 1F). The two are in a very good match. The match holds for all contrasts, as shown by the distribution of the difference of the predicted and simulated firing rate (Fig. 1F, inset). Note that to account for the refractory time of the neuron, 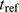, the final firing rate obtained from the linear rate model is corrected as 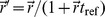 [22].

To better understand the mechanism underlying the operation of the network, we should look at the operator matrix, 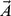, that gives the vector of stationary firing rates, as described in Eq. 14. Knowing the weight matrix, 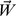, one can explicitly compute the operator matrix as 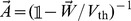. The emergence of selectivity and invariance is, therefore, essentially a linear function of the external input. As we have already previously discussed [21], two linear mechanisms are responsible for the emergence of high-fidelity orientation tuning: First we have a selective attenuation of the common mode, which means that the baseline component of the input is suppressed, but the orientation-specific component is not. In addition, random summation of functionally heterogeneous connectivity creates a distribution of neuronal tuning, which can lead to an amplification of neuronal selectivity (for details, see [21]).

#### Predicting the rectified responses of PIF networks

**Figure 2:**
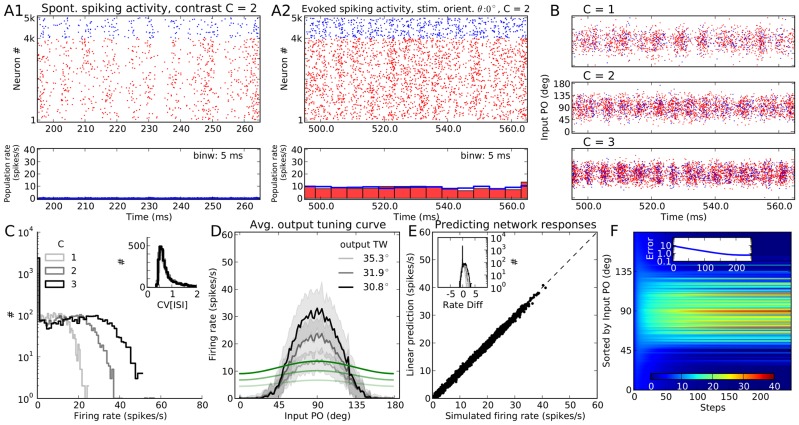
Population responses of the same PIF network with stronger inhibition. Same format as Fig. 1, for a network of PIF neurons with more recurrent inhibition (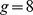), but otherwise identical parameters. (**A, B, C, D**) Same as panels (A, B, C, E) in Fig. 1, respectively. (**E**) The analytical prediction of the linear rectified model (Eq. (9)) is plotted vs. the results from numerical simulations. Distributions of the difference between the actual and the predicted firing rates (rate diff) at this orientation are shown in the inset for all three contrasts, respectively. (**F**) Firing rate of neurons during the iteration of the linear rectified rate equation Eq. 9. The equation is solved here for 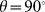 and for the medium contrast, 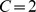. The x-axis shows the steps of the iteration (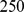 in total), for neurons sorted according to their input PO. For clarity, only 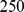 selected neurons are shown. Pseudo color indicates the firing rate of each neuron. In the inset, the evolution of the residual error is plotted. Error is defined here as the root mean square (RMS) of the difference between the actual firing rate vector and the firing rate vector at each step of iteration, i.e. 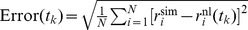. Here 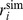 is the firing rate of neuron 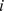 estimated from numerical simulations of spiking networks, and 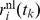 is the predicted firing rate of the same neuron at the *k*th step of iteration of the analytical equation.

Increasing inhibition dominance in our networks may lead to a nonlinear scenario where a part of the population turns silent. This can be obtained, for instance, by increasing the relative strength of inhibitory synapses, which is parametrized by 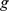. The result of the simulation of the same network topology as in Fig. 1, but with stronger inhibitory synapses (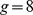), is shown in Fig. 2. The dynamic state of the network does not change, but the activity gets sparser (Figs. 2A–C), a natural consequence of having more potent inhibition in the network. As a result, now some neuronal firing rates get rectified, which is evident in the average population tuning curves (Fig. 2D): Neurons with input POs far from the stimulus orientation remain silent across all contrasts. This outcome corresponds to the prominent peak at zero in the distribution of firing rates in the network (Fig. 2C).

The straightforward linear theory, obviously, cannot account for the behavior of the network in this case, as zero-entries in the vector of firing rates also need to be consistent with the recurrent network dynamics. Instead, we solve an effective linear-rectified rate equation to compute the response of the network. A prediction based on Eq. 9 in the Methods section is plotted in Fig. 2E, demonstrating a very good match. The evolution of the firing rates to the final solution and its convergence are shown in Fig. 2F. Note that the refractory period is corrected for in the same manner as described in the previous section.

#### Predicting responses of LIF networks

**Figure 3:**
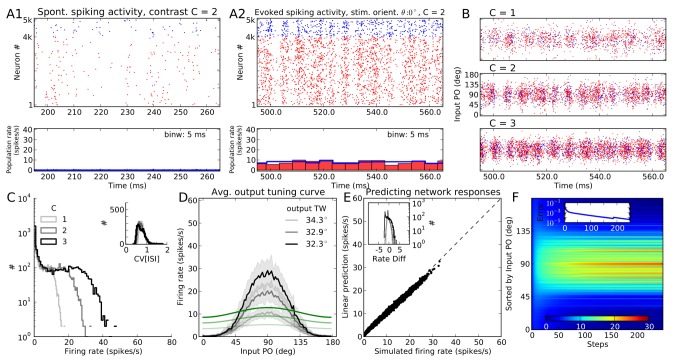
Population responses of a network of LIF neurons to an oriented stimulus. Same as Fig. 2, for the a network with the same parameters, but composed of LIF instead of PIF neurons.

To compare our results on networks of PIF neurons with networks of LIF neurons, we keep all the parameters (in particular the connectivity matrix) the same as in the previous section (i.e. 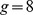), and only change the neuron model. The results are shown in Fig. 3. Again, the network operates in the AI state, and the LIF responses look indeed very similar to the PIF case. The only noteworthy differences are slightly smaller output firing rates and more irregular spike trains (Fig. 2C vs. Fig. 3C), accompanied by a less prominent rectification (compare Fig. 2D and Fig. 3D). This is due to a different input-output transfer function of the neuron model: Whereas a PIF neuron responds only to the net mean input current, a LIF neuron is sensitive to both the mean and the variance of the input current (see Methods).

The slightly more complicated transfer function of the LIF neuron makes a prediction of the neuronal responses using the linear rectified equation in the previous section incomplete. We therefore resort to an extended version of the equation where the transfer function of the neuron (an approximate solution of the first-passage time problem) is explicitly included. We now have to solve an effectively non-linear fixed-point equation involving the firing rates of all neurons (see Eq. 13 in Methods). The result of this is shown in Fig. 3E, and the convergence of the result is illustrated in Fig. 3F. The excellent match of our results throughout our study suggests that it is possible to correctly predict the neuronal responses of the network even for the nonlinear scenario implied by neurons with a membrane leak. Adding a leak and keeping the input fixed lead to a minor reduction of the firing rate of neurons in the network, as described above. One might therefore ask what happens if the external (feedforward) input is increased so that neurons in the LIF network have the same firing rates as in the PIF network. Numerical simulations were performed to address this question directly. Indeed, very similar orientation selective responses were also observed in this case, at all contrasts (S3 Fig.), further underlining the robustness of the results reported here. A very small difference, however, can be reported for TW. Whereas a comparable TW was obtained for PIF and LIF networks at the lowest contrast (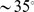), a subtle sharpening of TW for PIF networks was observed at higher contrasts (S3 Fig.). This is again a result of the sharp onset of rectification in PIF neurons, in contrast to the somewhat smoother transfer function of the LIF neuron model (see also below, for further discussions).

### Computing individual tuning curves

**Figure 4:**
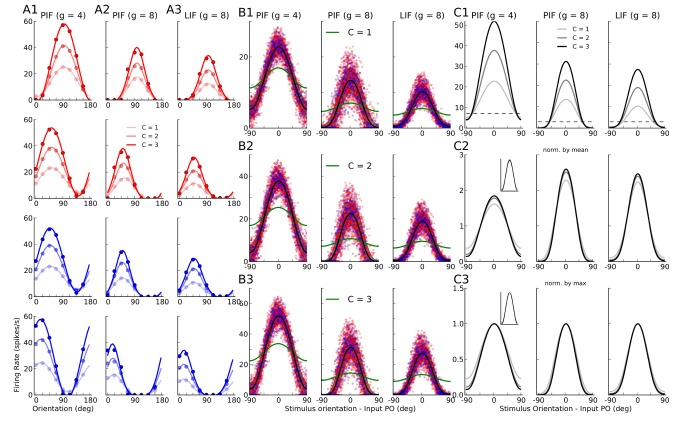
Orientation selectivity in networks of PIF and LIF neurons. (**A**) Sample tuning curves for two excitatory (red) and two inhibitory (blue) neurons at three different contrasts. For otherwise the same set of parameters, the tuning curves are extracted from neurons in the PIF network with 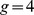 (A1; same as Fig. 1), the PIF network with 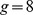 (A2; same as Fig. 2), and the LIF network with 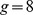 (A3; same as Fig. 3). Dots are the results from numerical simulations, and the lines show the best fitting von Mises function (see Methods for details). Lighter colors correspond to lower contrasts in each case. (**B**) Data from a sample of 10% of all neurons in the network are aligned to the preferred orientation of the input, respectively, and plotted for different contrasts. The solid line is the tuning curve obtained from averaging over all 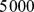 neurons, and the shading shows the standard deviation. The range of input POs is broken into 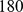 bins, and mean and STD are then computed from all neurons falling into the same bin. The green line shows the feedforward input, normalized to have the same mean value as the output tuning curves. The plots corresponding to different contrasts are shown in separate rows, with the top panel (B1) corresponding to the lowest contrast (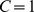), the middle panel (B2) corresponding to the middle contrast (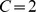), and the bottom panel (B3) corresponding to the highest contrast (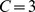). (**C**) Average tuning curves from (B) are shown in the top panel (C1) for all contrasts, with lighter colors corresponding to lower contrasts. The dashed line shows the level of spontaneous activity, i.e. the background firing rate in absence of feedforward stimulation (this is the term 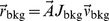 in Eq. 14). In the bottom panels, the tuning curves are compared after normalization, first by normalizing each tuning curve to its mean (middle row, C2), and then by normalizing it by its maximum value (bottom row, C3). In the inset of the first column, the normalization is performed after subtracting the spontaneous activity.

To better study the output tuning properties of neurons in the network, we extract individual tuning curves from simulations. This is obtained by repeating the previously described procedure for all 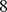 different stimulus orientations. The output tuning curve of a neuron 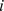, 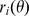, is then extracted as its stationary firing rate in response to each orientation. Sample tuning curves for randomly picked neurons are shown in Fig. 4A, for all three types of networks discussed before. Similar to average tuning curves, individual neurons show strong output responses for a narrow range of orientations, and this selectivity remains stable across different contrasts.

The tuning curves are similar for the networks of LIF and PIF neurons with 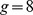, respectively. Again, the only noteworthy differences are sharper rectification for orthogonal orientations, and slightly higher firing rates in networks of PIF neurons. The PIF network with less inhibition (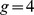) shows higher activity and almost no rectification. These properties are more evident when we look at more tuning curves from the networks, aligned to their input POs (Fig. 4B): The smoother input-output function of the LIF neuron manifests itself in a less abrupt suppression of firing activity at orthogonal orientations, and the mean output tuning curve of the network has a slightly lower amplitude than the same tuning curves in a PIF network with 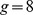 (Fig. 4B and Fig. 4C, upper panels). The PIF network with less inhibition again shows the strongest activity.

However, all the networks show similar properties regarding OS: Strong and highly selective output responses are obtained as a result of recurrent network operation, and this has the same profile for all contrasts considered (Fig. 4C, upper panel). In fact, normalizing the mean tuning curve of the network across different contrasts yields virtually indistinguishable tuning curves (Fig. 4C, lower panels), similar to what is reported in experiments [23]. Note that the worst contrast invariance is observed for the PIF network with 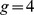 (the first column). This is the result of a rather high spontaneous activity level, i.e. the activity of the network to background input (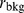), in absence of an orientated stimulus. This activity is the same for all contrasts, as it is stimulus independent. If these spontaneous responses are subtracted (similar to the preprocessing some experimentalists perform [3]), the resulting normalized tuning curves now show a perfect contrast invariance (Fig. 4C, Inset).

Although quite similar in general, the normalized tuning curves reveal a minor deviation from perfect contrast invariance: the average tuning curves are slightly broader at the lowest contrast (see also the results below). Increasing contrast in our networks, therefore, even leads to a small enhancement of orientation selectivity (for a similar experimental observations, see [8], [24]. Note that this is the opposite of what is expected from the so-called “iceberg effect”, which implies a broadening of tuning curves for higher contrasts (for a similar observation, see [20]). In our networks, this is mainly a consequence of potent rectification at higher contrasts. This is explained in more detail below.

It is important to note that the sharp tuning of inhibition we observed here (Figs. 1–4) is not a necessary ingredient for the other results. As we have assumed completely symmetric feedforward and recurrent properties for excitatory and inhibitory neurons in our networks, a comparable level of output responses is a necessary consequence. In biology, however, excitatory and inhibitory neurons exhibit different responses. This is caused by different single cell properties, like different spike thresholds and response patterns, as well as different wiring patterns of feedforward and recurrent connections. As a result, different tuning properties would be expected for inhibitory neurons. In fact, different experimental results have been reported for different subtypes of inhibitory neurons [25], [26]. We therefore tested whether our general results still hold if inhibitory neurons show broad tuning, and we found that all results are retained under such conditions (S1 Fig.). Thus, tuning properties of individual neurons do not alter our results, and the role of inhibition for the emergence of OS remains unchanged in our inhibition-dominated networks.

#### Distribution of orientation selectivity

**Figure 5:**
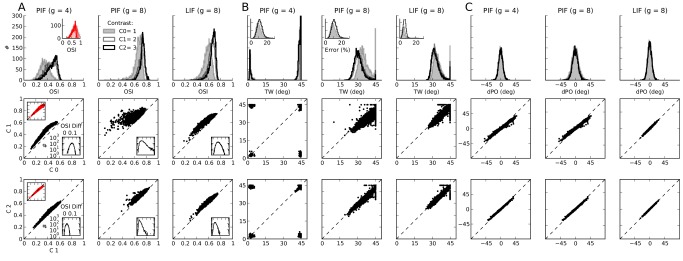
Distribution of orientation selectivity in all three networks considered. (**A**) Distribution of the orientation selectivity index (OSI, see Methods) for networks of PIF and LIF neurons. Lighter colors indicate lower contrasts, and the distribution for the lowest contrast is filled for better visibility. For the network of PIF neurons with 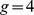 (first panel), the distribution of OSI for tuning curves after subtraction of spontaneous activity is plotted in red in the inset. In the bottom panels, the OSIs of individual tuning curves are compared at different contrasts: The OSI at the middle contrast is plotted vs. the lowest (middle row), and the OSI at the highest contrast is plotted vs. the middle (bottom row). For the first column, the same plot is shown for the OSI obtained after subtracting spontaneous activity (inset top left, red). Also shown is the distribution of the difference in the OSI between the two contrasts in each case (inset bottom right). (**B**) Distribution of tuning width (TW) in the networks for different contrasts. TW is computed from the best fitting von Mises function to individual tuning curves (see Methods). The error of this fit is quantified by an error index in percent (see Methods), the distribution of which is plotted in the inset. In all panels in this figure, only the results from tuning curves with an error smaller than 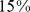 (at all contrasts) are plotted. The bottom panels show the scatter plot of TW at different contrasts, similar to the bottom panels in (A). (**C**) Same as in (A) and (B) for the difference in input PO and output PO of individual neurons (dPO).

To study the degree of OS across the population, we compute the preferred orientation (PO), orientation selectivity index (OSI) and tuning width (TW) of tuning curves of individual neurons (see Methods for details). The distributions of OSI, TW and the difference between output and input PO (dPO) are shown in Fig. 5 for different contrasts. In general, a high degree of OS, narrow TW and a small scatter dPO are obtained across the population, in accord with our observations documented in Fig. 4. Moreover, this result is consistent for networks of LIF and PIF neurons, with and without rectification.

The contrast invariance of all these properties can already be expected from the stability of the distributions for different contrasts. Responses to preferred orientations are stronger at higher contrasts (Fig. 4), and the overall measures of OS are quite stable. This can be demonstrated further by inspecting OSI, TW and dPO of individual neurons at high contrasts and low contrasts (Fig. 5, lower panels). Very low PO scatter in each case corroborates the visual inspection of contrast invariance. The strongest deviations are again obtained for the PIF network with low inhibition (Fig. 5A, the first column). If we again subtract the spontaneous activity, as described before, perfect contrast invariance is obtained also in this case (Fig. 5A, insets in red color, first column).

In general, however, the contrast invariance improves for higher contrasts, i.e. the highest contrast vs. the middle contrast values for OSI and TW lie closer to the diagonal compared to the middle contrast values vs. the lowest contrast. This is in line with our previous observation (see Fig. 4) that the average tuning curves of the network show better contrast invariance for higher contrasts. Furthermore, the enhancement of tuning curves for higher contrasts is clearly visible here. The distribution of OSI is shifted towards smaller values, implying an enhancement of OS for higher contrasts.

Likewise, the distribution of TW (Fig. 5B, upper panels) is shifted towards larger values for the lowest contrast, i.e. an overall — although small — sharpening of tuning curves upon increasing the contrast is resulting. This effect is mainly visible in the middle column, i.e. for the PIF network with strong inhibition. This is the network with the strongest rectification: The PIF network with 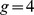 (first column) has a lower inhibition dominance ratio, and as a result, the high level of spontaneous activity keeps the tuning curves away from rectification. The network of LIF neurons with 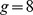 (third column) is equipped with another mechanism, namely a smooth transfer function of the LIF neuron due to its general sensitivity to fluctuations. As a result, with otherwise the same set of parameters, fewer neurons are strongly rectified.

The size of the peak of the TW at 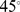 is another indication of the underlying mechanism in each case: The PIF network with 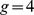 has the strongest peak at this value of the TW. This is in fact a sign of the linear operation of the network, as a purely linear network does not distort the cosine tuning of the input and generates a clean cosine output tuning curve. There is no sign of a significant sharpening of the tuning curves (reducing the TW), consistent with the linear operation of the network.

For both PIF and LIF networks with 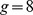, a distribution of TW results, with tuning curves as sharp as 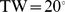. There is, therefore, some nonlinearity in these networks, which sharpens input cosine tuning curves with 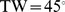. However, there is still a residual peak at 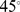, reflecting the fraction of neurons for which the contribution of nonlinear mechanisms is very small. Note that this peak changes differently for networks of PIF and LIF neurons: For the PIF network, at the lowest contrast, a large number of tuning curves have 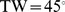, i.e. they do not get significantly rectified. This is because the output modulation for a significant fraction of the population is not yet strong enough to surpass the output baseline firing rate.

At higher contrasts, this fraction becomes much smaller, since the strong output tuning curves imply a significant degree of rectification for the majority of the population. As a result, both a sharpening of tuning curves and an enhancement of OSI, but not a significant change in POs, are obtained. Once the negative halfwave of the cosine is fully rectified, because the baseline firing rate is too low, there is no room for even more rectification, explaining the stability of TW and OSI distributions for higher contrasts. For LIF networks, however, this transition between low and high contrasts is smoother. This is again due to a smoother input-output transfer function of the neuron model, which implies a less strict rectification.

This result can be summarized by plotting the distribution of tuning widths for all three networks considered here, for each contrast (S2 Fig.). It is clear that the PIF network at 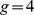 has the most linear input-output relation, which leads to a low degree of sharpening of tuning curves (S2A Fig.). PIF and LIF networks with more inhibition (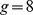), in contrast, show a significant amount of sharpening, and this is a robust phenomenon at all contrasts (S2B, C Fig.). This phenomenon can be further quantified by a sharpening coefficient ranging between 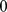 and 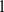 (S2D–E Fig.), where the highest sharpening corresponds to values close to 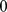. The stability of the distributions at different contrasts again reveal the contrast-invariance of tuning widths.

#### What determines individual tuning curves?

**Figure 6:**
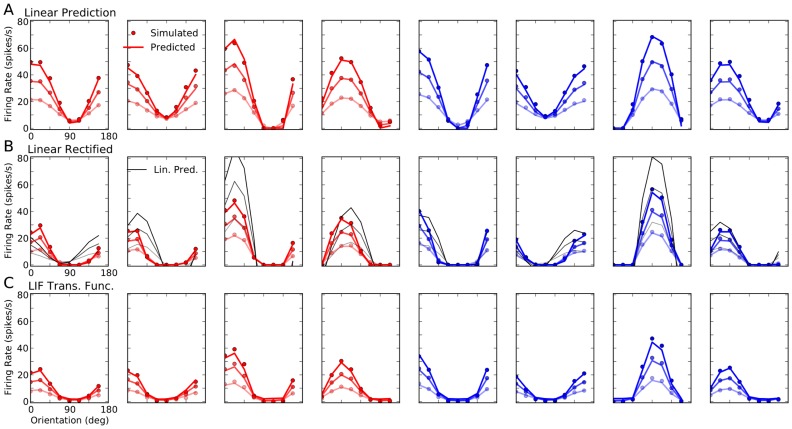
Analytical computation of individual tuning curves for different networks. Output tuning curves extracted from numerical simulations (dots) for a sample of 12 excitatory (red) and 12 inhibitory (blue) neurons are compared with the corresponding analytical predictions (lines), based on the known properties of the neuron model used and the exact network topology. In (**A**) we used the linear theory (from Eq. (14)), in (**B**) the linear rectified model (from Eq. 9), and in (**C**) the nonlinear input-output rate equation for LIF neurons (from Eq. 13). In panel (B), the result of a linear prediction (from Eq. (14), assuming no rectification) for 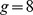 is plotted in black lines, for comparison.

**Figure 7:**
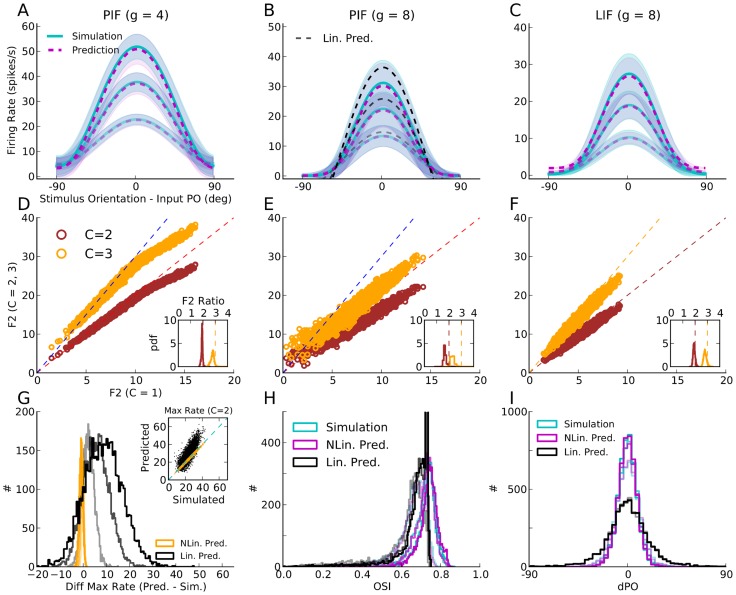
Average tuning curves in different networks. For the network of PIF neurons with 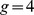 (**A**) and 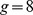 (**B**), and for the network of LIF neurons with 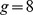 (**C**), the average tuning curves are shown for comparison. Individual tuning curves are aligned to their Input PO, and the mean and standard deviation of all data points falling in bins of size 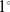 are computed, separately for each contrast. The average tuning curves extracted from simulations are plotted as solid lines (cyan), and the dashed line (magenta) shows the average tuning curve from analytical predictions. The shading indicates the standard deviation, in respective colors. In (B), the average aligned tuning curve from a linear prediction is also plotted for comparison (black dashed lines). Lighter colors correspond to lower contrasts. (**D, E, F**) For each neuron, the modulation (F2) component of the tuning curve is computed at each contrast. This value for higher contrasts (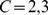) is then scattered against the lowest (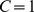), respectively. The dashed lines denote the result of linear amplification of modulation, 
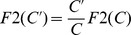
. The distributions of the respective modulation (F2) ratio 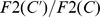 are plotted in the inset, respectively. (**G**) In PIF networks with 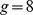, the maximum firing rates of individual tuning curves, 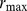, are computed. The difference of this maximum value from the simulated results (Diff Max Rate) is then computed (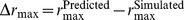). The distribution of this difference is plotted for different contrasts, for the linear rectified prediction (NLin. Pred.) and for the linear prediction (Lin. Pred.). Inset: For the middle contrast (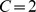), 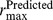 is plotted vs. 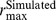, for both predictions. (**H, I**) For the PIF network with 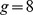 and for different contrasts, the distributions of OSI and dPO (output PO - input PO) are plotted, respectively. For the simulated results and for the linear rectified prediction, the distributions show almost a perfect match. This is compared with the linear prediction (in black).

In Figs. 1–3, we analytically computed the population responses of the three different networks to one specific orientation. If we now repeat the same procedure for all orientations, we can analytically determine the shape of each and every individual tuning curve. The predicted tuning curves for some sample neurons are compared with the result of numerical simulations in Fig. 6. The shape of the average tuning curves of the three networks in question is shown in Fig. 7, A–C, respectively. Again, the result of the analytical theory matches very well the outcome of numerical simulations, corroborating the fact that our linear and nonlinear rate models capture all essential properties of OS in the networks considered here. When it comes to explaining how OS emerges in these networks, and which mechanisms determine its properties, we therefore tend to believe that our analysis is complete.

#### Mechanisms underlying orientation selectivity

Comparing the resulting tuning curves in the different networks can cast further light on the mechanisms underlying OS in each case.

First, from the linear operation of the network, there are already two properties evident (Fig. 6A): One property is that, while the baseline component of the tuning curves is selectively suppressed, the tuned component is maintained or even enhanced; for further explanation of this process, see [21]. Another important property is that the tuning curves have essentially the same shape at different contrasts. In particular, they have their minimum and their maximum at very similar orientations.

Note that the latter property follows directly from the linearity of the operator 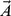, if the term 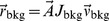 in Eq. 14 is negligible or subtracted (with the parameters used here, 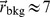 or 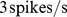, for 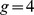 or 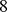, respectively).

These linear properties, leading to highly selective and contrast invariant output responses, are mostly preserved in nonlinear networks. There are however some additional mechanisms, which change the shape of output tuning curves, and the processing of selectivity (Fig. 6B, C). The most immediate consequence of nonlinear processing is a sharpening of the tuning curves (Fig. 5B), as discussed before. Another property is a global attenuation of tuning curves, without distorting them. In fact, the linear theory overestimates the amplification of tuning curves. This is shown in Fig. 6B for the PIF network with 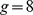, where the result of the linear prediction (obtained from Eq. 14) for this network is compared with the solution of the linear rectified rate equation that is directly obtained from Eq. 13.

This discrepancy is further demonstrated in Fig. 7B, where the average tuning curves of the network at different contrasts are compared with the predictions by different theories. Although linear theory predicts the mean and the standard deviation of overall tuning curves quite well for the PIF network with 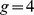 (Fig. 7A), it is generally overestimating the tuned output firing rates for the PIF network with strong recurrent inhibition (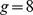, Fig. 7B). The discrepancy is strongest for neurons that are stimulated at their preferred orientations, at higher contrasts, consistent with the visual inspection of tuning curves (Fig. 6B).

To identify the source of this discrepancy, we consider the amplification of modulation (F2) component at different contrasts. In Figs. 7D–F, the F2 components of individual output tuning curves are extracted. The F2 components at higher contrasts (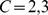) is then scattered against the F2 component at the lowest contrast (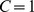). If the network operation was linear, increasing the contrast should only scale the modulation. This is indeed almost the case for PIF network with 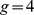 (Fig. 7D), where most of the dots lie on the dashed lines, denoting a linear amplification. For neurons with large F2 components, however, the linear relation breaks, and a sublinear amplification is observed. This means that the nonlinearity of rectification is only effective for strongly tuned inputs: Their F2 components surpass the F0 component, leading to a partial rectification of tuning curves.

In the PIF network with 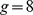, in contrast, most of the neurons deviate from the behavior predicted from linear amplification (Fig. 7E). This is consistent with our previous observation that a significant degree of rectification exists here. Interestingly, the LIF network with 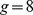 shows the weakest deviation from the linear behavior (Fig. 7F). This is again in line with our observation that LIF networks do not exhibit all-or-nothing rectification, as a result of the smoother input-output transfer function of their neurons. As all neurons are responsive even to subthreshold input, effectively very little rectification is observed. This explains the surprisingly good match of the LIF network with the predictions from a linear theory.

An explanation for the suppression of the modulation component can be given as follows: As a result of rectification of firing rates, baseline and modulation are not processed independently any more. In fact, the rectified population tuning curve now assumes a component that has a non-zero projection in the direction of the common mode. As a result, the rectified modulation vector also hits the suppressed mode of the network. The network does not allow the modulation to grow without bounds, and a suppressive mechanism controls it by a process which very much resembles normalization: The stronger the tuned component is, the stronger is the negative feedback and the corresponding divisive suppression, which was previously selective to the untuned component.

In general, the linear theory predicts higher firing rates in average tuning curves (Fig. 7B), it overestimates the peak of tuning curves (Fig. 7G), it leads to smaller OSIs (Fig. 7H) and a larger PO scatter (Fig. 7I). The decrease in the maximum firing rate due to non-linear processing (Fig. 7G) is a consequence of the suppression of modulation. The suppression is more prominent for strongly tuned neurons with more pronounced modulation components. The deviation in the peak activity is more pronounced for high amplitudes, and small tuning curves behave almost linearly (Fig. 7G, Inset). The OSI is enhanced by rectification (Fig. 7H), as this leads to a complete suppression of responses at non-preferred orientations. Finally, the rectifying nonlinearity brings about less deviation of output from input POs. The linear prediction of neuronal responses deviates more from the input signal and results in a wider distribution of PO scatter than observed in simulations (Fig. 7I).

This result may look counter-intuitive, as the distortions introduced by a nonlinearity in output tuning curves are in fact inducing less distortion in feature selectivity, enhancing the fidelity of the network in representing the input signal. An intuitive account of this process can be given in terms of the effective number of active neurons. Rectification of firing rates decreases the effective population of neurons contributing in the recurrent network. As the degree of PO scatter in the network depends on the random summation of presynaptic POs [21], an effectively smaller population of presynaptic neurons leads, on average, to a smaller change in output POs, assuming they represent an independent sample.

### Orientation selectivity is robust across different regimes

#### Asynchronous vs. synchronous network dynamics

We have shown that OS emerges robustly in networks of both PIF and LIF neurons, with and without rectification. We also have shown that the firing rate of every individual neuron, along with all properties of OS, can actually be analytically computed in these model networks. We now ask the question, if these results and their underlying mechanisms are robust across different regimes of network dynamics. In particular, is the state of network dynamics crucial for OS and CI to emerge in our networks?

Recurrent networks of excitatory and inhibitory spiking neurons are known to exhibit a rich dynamic repertoire, ranging from asynchronous to synchronous states, a property that they share with the mammalian neocortex [27]. One may therefore ask, if the state of neuronal network dynamics influences its function in a crucial way. For instance, is it necessary for the networks studied here to operate in the AI regime?

**Figure 8:**
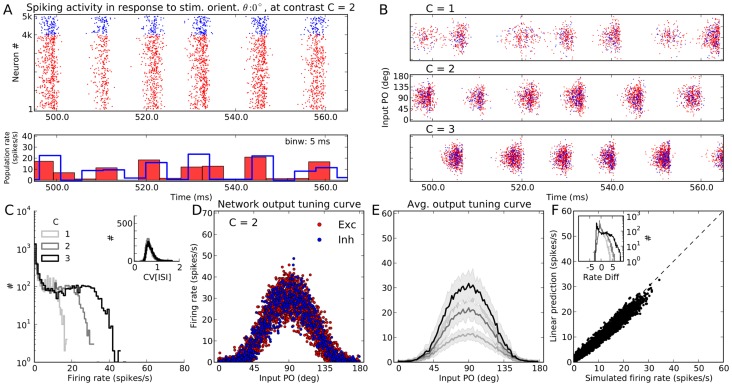
Population responses of the network of LIF neurons in a synchronous state. All panels and conventions are the same as Fig. 1. The network parameters are the same as Fig. 3 (network of LIF neurons with 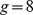). Only the transmission delays of recurrent synapses are now all the identical, 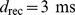, in contrast to the random distribution of delays used in Figs. 1–3. Simulation of each stimulus orientation is run for a shorter time of 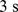, leading to a somewhat larger scatter in panel (F).

In fact, with the constellation of parameters we work here, with strong external input and inhibition dominating the recurrent network, we may easily end up in a highly synchronous state with fast oscillations, if we do not impose a broad distribution of synaptic transmission delays, for instance. A raster plot of activity for a LIF network with identical connectivity, and with 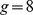, but with a delay of 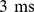 for all synapses is shown in Fig. 8A. Although the activity state looks very different, the orientation tuning looks very similar to what we obtained for the AI network, shown in Fig. 3. In both cases, exactly the same nonlinear input-output transfer function predicts the feature specific responses quite well (compare Fig. 3F with Fig. 8F).

**Figure 9:**
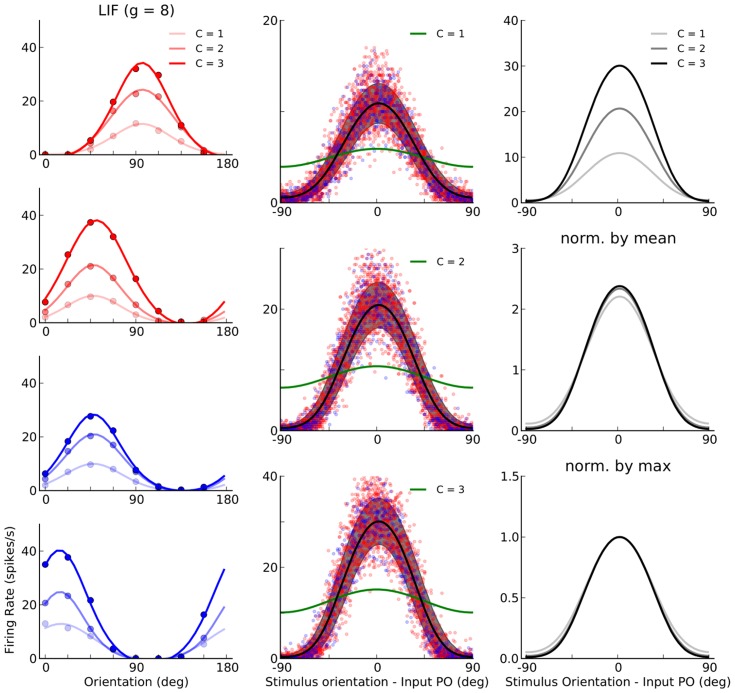
Orientation selectivity in a synchronous state of network dynamics. Same as Fig. 4, for the networks of LIF neurons with a fixed transmission delay of recurrent synapses, 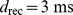. This brings the networks to operate in a fast-oscillating synchronous state.

Consistent with the similarity of population tuning curves, tuning of individual neurons also shows a very similar behavior across regimes (Fig. 9). Strong responses at orientations close to the input PO are obtained at all contrasts, indicating the emergence of highly selective and contrast invariant output tuning curves.

The dynamic state of the network, thus, does not compromise OS and CI of output responses, and leads only to very subtle differences. This outcome could have been expected, as it is based on average firing rates and, as such, does not depend on the details of temporal dynamics in the network.

#### Mean-driven vs. fluctuation-driven activity

Balanced networks with strong synapses are known to settle in a fluctuation-driven regime of activity, where the mean input to neurons remains below threshold on average, and only temporal fluctuations lead to spiking activity [28]. It is therefore important to ask to which degree such a regime of activity is necessary for the network to maintain its highly selective output responses, with contrast invariant tuning curves.

Considering PIF neurons here helps to elucidate the contribution of mean and fluctuations. As opposed to LIF neurons the firing rate response of which depends on the first two moments of the total input to the neuron, the firing rate of PIF neurons only depends on the mean input. In fact, we used this property to derive a linear rate equation (Eq. 6), which describes the operation of the network merely in terms of the mean values of (excitatory and inhibitory) input and output firing rates. All the results that we have presented here for networks of PIF neurons are therefore valid for a mean-driven regime of activity as well.

We conclude that fluctuation-driven activity is not a prerequisite to obtain strongly tuned and contrast invariant neuronal responses. Likewise, all the linear mechanisms of the network, like selective suppression of the common-mode and selective amplification of modulation, as well as nonlinear ones, like sharpening and normalization, are perfectly compatible with both fluctuation-driven and mean-driven activity.

**Figure 10:**
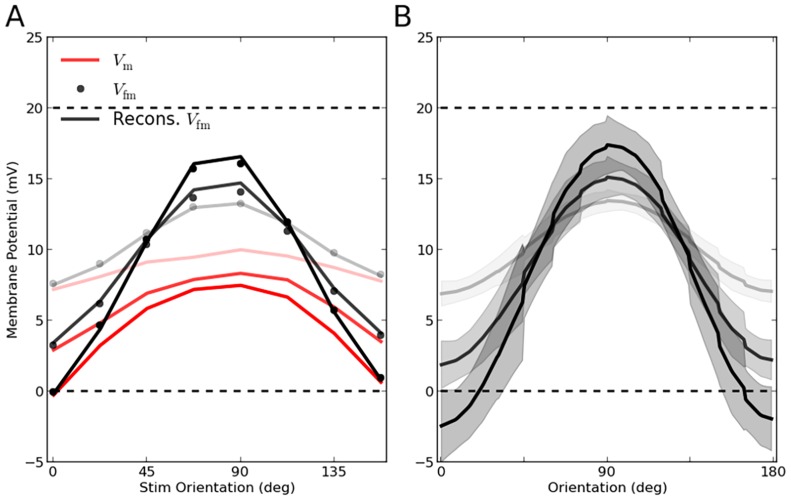
LIF networks operating in a fluctuation-driven regime of activity. Tuning of the membrane potential for neurons in the LIF network shown in Fig. 3. (**A**) The time-averaged membrane potential is shown for a sample excitatory neuron with input PO 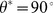, at different orientations and different contrasts of the stimulus. Lighter colors correspond to lower contrasts, respectively. 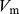 (red lines) is the actual membrane potential, and 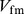 (black dots) is the free membrane potential. The free membrane potential is obtained by correcting for the net hyperpolarization resulting from voltage reset after each spike, i.e. 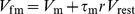. The reconstructed free membrane potential (black lines) is the prediction from the net input to the neuron, including the background input, 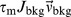, the feedforward input, 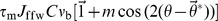, and the recurrent input, 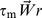. (**B**) Average free membrane potential for more sample neurons, 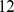 excitatory and 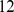 inhibitory, with different input POs. All tuning curves are aligned and averaged across bins of size 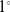. The mean tuning curve is plotted as solid lines, and the shading shows the standard deviation across neurons.

This can be investigated further by analyzing the LIF simulations in more detail. In fact, the mean membrane potential of LIF neurons remains always below threshold (Fig. 10). Neurons in the LIF network, for all contrasts and at all orientations, are therefore operating in the fluctuation-driven regime. Nevertheless, the properties of OS in these networks were pretty much the same as mean-driven PIF networks. The results and the analysis we provided here are therefore general, and do not depend on a specific dynamic regime of activity.

## Discussion

We studied the mechanisms contributing to the emergence of orientation selectivity in randomly connected networks of spiking neurons that lack any feature specific connectivity. The OS of neurons in rodent primary visual cortex is very similar to what we see in numerical simulations of networks of integrate-and-fire neurons, and our subsequent analysis revealed both linear and nonlinear mechanisms underlying these properties. Our linear rate-based account of the emergent properties of such sensory networks captured two essential mechanisms: selective suppression of the common mode, and selective amplification of the feature specific component. Together these two yielded highly selective output responses of most neurons in the network, and it fully accounted for the contrast invariance of the tuning curves.

According to our analysis, rectification of negative firing rates was the single most important source of nonlinearity in our system. An extended linear theory was able to fully account for the distortions introduced by this nonlinearity in output tuning curves. Interestingly, this nonlinearity also lead to a reduced distortion in the representation of the feedforward signal, i.e. the output POs of neurons differ less from their input POs. Moreover, a general sharpening of tuning curves was induced by this nonlinearity. This is consistent with experimental findings on the role of the spiking nonlinearity in sharpening the membrane potential tuning [29].

Another consequence of the nonlinearity was a suppression of the feature specific component, which was more effective for neurons with higher output modulation. The suppressive mechanism of the network, which was selective to the common mode in the linear regime, is now controlling also the modulated component, provided the nonlinearity is strong. In this scenario the tuned component cannot grow beyond all bounds. If the suppressive mechanism is the same as the divisive attenuation of the common mode [21], it should in fact act as a divisive normalization of the modulation. Computational analysis of this mechanism is beyond the scope of the current article and needs further study.

The results were robust across different states of network dynamics, different regimes of activity, and for different neuron models. Networks operating in the AI state, as well as in a synchronous state with fast oscillations, showed the same degree of OS. Mean-driven PIF networks exhibited essentially the same neuronal tuning as networks of LIF neurons settled in a fluctuation-driven regime of activity. We could also rule out a significant contribution of the leak in networks of LIF neurons, another nonlinear mechanism described in more detail in a previous study [21].

We expect that the same mechanisms are at work, and hence a similar analysis would predict the emergence of OS and its properties, in models with more heterogeneous connectivity, and with more complex neurons (as in [19]). It is, however, possible that additional nonlinear mechanisms, like a power-law transfer function [30], [31], play an additional role in other networks. It may, however, be quite difficult to tease apart the contribution of each of the underlying mechanisms. By predicting the exact output tuning curves in our networks, composed of different flavors of integrate-and-fire neurons operating in very different regimes of inhibition dominance, dynamics and activity, we were able to pinpoint the main contributing mechanisms to OS. Pursuing the same analysis strategy for other networks could help to elucidate the exact nature of underlying mechanisms in each case.

Our rate-based description of network dynamics here completely ignored a possible contribution of pairwise correlations across neurons. This was in spite of a not particularly sparse connectivity of excitatory neurons, and an even denser connectivity of inhibitory neurons. The effect of the shared input, however, did not manifest itself in the firing rates, and in fact our predictions worked quite well. A possible additional contribution comes from the fact that the network is dynamically decorrelating itself, as a result of balance of excitation and inhibition, as has been demonstrated before [32], [33]. Dense pattern of inhibitory connectivity in local networks, as we have used here and has been reported experimentally in different cortices [12], [34], [35], might therefore further enhance this decorrelation.

Inhibition dominance was also crucial for an optimal operation of our networks in achieving highly selective and contrast-invariant orientation-specific responses. Note that this inhibition was provided by recurrent inhibition in our model networks, and purely feedforward inhibitory circuitry would not support the mechanisms we described here (for a more elaborate discussion, see [21]). It has been reported in experiments that broadly tuned inhibitory input contributes to sharpening and contrast-dependence of OS responses in mouse visual cortex [24], [36], and it has been suggested that the feedforward inhibition resulting from broadly tuned PV expressing interneurons is the underlying mechanism [24], [36]. Our explanation here suggests instead that the resulting contrast-dependent, untuned inhibition is an emergent property of the network, and not of individual neurons. That is, even if individual inhibitory neurons are themselves sharply tuned (as illustrated e.g. in Fig. 4), the resulting recurrent inhibitory input each neuron receives within the network can be untuned.

Note that this does not mean either that tuned inhibition is necessary, as our model behaves essentially the same way when inhibitory neurons are only poorly selective (S1 Fig.). Strongly tuned as well as weakly tuned inhibitory neurons [25], [26] can equally well serve the role of recurrent inhibition in shaping OS responses and leading to contrast-invariance. What matters is that in both cases the net inhibitory input, as a result of recurrent interactions within the network, becomes untuned and proportional to the average activity of the excitatory population, such that a contrast-dependent untuned inhibition results as an aggregate property of the network dynamics, irrespective of sharp or broad tuning of its inhibitory neurons.

The above scenario can also explain why dark-reared mice, which lack a broadening of PV+ responses, still operate with untuned aggregate inhibitory input from the network and hence show highly selective responses [36]. As we demonstrated with our simulations and analyses here, selectivity of output responses does not depend on the (un)selectivity of individual inhibitory neurons. Rather, it is the interaction of excitation and inhibition in the recurrent network that determines the output selectivity. Our model is, therefore, fully consistent with the findings by [36], who demonstrated experimentally that “blocking the broadening of output responses of individual inhibitory neurons does not block the broadening of the aggregate inhibitory input to excitatory neurons”. Our model also explains the results of a previous experimental report demonstrating that “broad inhibitory tuning” of fast spiking cells is “not required for developmental sharpening of excitatory tuning” [37]. Based on these convergent results, we therefore hypothesize that untuned inhibition is an emergent property of an inhibition-dominated network, and not necessarily a result of the feedforward action of broadly-tuned fast spiking interneurons.

Our simulations demonstrated that the LIF network showed the “best” contrast-invariant behavior (see Fig. 5) and may thus provide a particularly good match with biology. This observation is, in fact, consistent with previous experimental observations on the role of noise for contrast-invariance of orientation tuning [23]. In contrast to the PIF neuron model, the LIF neuron model has a smooth input-output transfer function, which in turn seems to enhance the contrast-invariance of output responses [23]. That is, as opposed to the PIF model with a sharp all-or-none threshold, in the LIF model even “subthreshold” inputs can elicit some low-rate spiking activity. Our use of PIF and LIF neuron models in different operating regimes, therefore, also sheds light on this aspect of orientation selectivity, in a realistic network model showing OS responses.

The contrast-dependence of OS responses that we observed in our networks (see Fig. 5) can also be linked with experimental findings. In fact, in layer 4 of mouse visual cortex, such contrast-dependence of OS has recently been reported [24], with a decrease in TW and an increase in OSI upon increasing the contrast. This is in line with our results, illustrated in Fig. 5. Such a contrast-dependence of OS was also reported previously in ferret primary visual cortex [8], where an inverse relation between the circular variance (that is 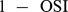) and stimulus contrast was reported.

Our results and analyses here suggest a satisfactory explanation for this puzzling combination of contrast-invariance and contrast-dependence of OS. First, the general invariance with regard to contrast is a natural consequence of the fact that OS is processed by inhibition-dominated networks. This is a result of the linear recurrent network operation, which suppresses the baseline and counteracts the iceberg effect, thus generating neuronal selectivity across different contrasts. The nonlinear properties of single-neuron transfer functions can now act on top of this and even enhance OS further when contrast increases. Contrast-invariance is primarily the result of linear network operation, whereas the subtle contrast-dependence came as a consequence of nonlinear neurons.

To zoom in on the role of single-neuron properties and different regimes of network dynamics, we intentionally neglected the role of specific structure in network here and rather confined our study to random networks without any specific connections. A more realistic assumption would be to consider networks with distance-dependent connectivity, which is what we find in the biological cortex. Notably, the same results as those obtained for totally structureless random networks have also been reported in networks with some structure, given certain conditions on the stability of the balanced state [19], [38], [39]. Specifically, it has been shown that the same kind of analysis we performed here can be extended to account for all properties of OS in such networks, as long as the extent of recurrent inhibition is more local than that of excitation [38]. This condition has in fact been derived analytically in [39], demonstrating that models of OS based on a sharper tuning of recurrent excitatory input (as e.g. considered in [40], [41]) cannot be consistent with the stability of the AI state. Remarkably, in the latter models a high level of pairwise correlations also arises [42], [43], which is not consistent with the generally low pairwise correlations reported in the input layers of visual cortex [43], [44].

We also ignored feature-specific connectivity [11]–[15], another biologically reported structure which arises later during development, and which should be considered in future modeling studies. Linear amplification of feedforward tuning has, for instance, been recently reported in rodents [16]–[18], presumably as a result of such feature-specific connectivity. In such a case, the above-mentioned issues with regard to the stability of the balanced state as well as the emergence of large pairwise correlations will be again posed, as now the same structure would exist in a functional space (rather than in the physical space of neuronal connectivity). How those issues could be dealt with by the cortex in the functional domain, and what further functional properties are resulting from such a structure, are interesting questions that ask for further investigations.

We conclude with a note on the solution of nonlinear rate equations in the network. To obtain the activity of each neuron in the network in the presence of a nonlinearity (rectifying nonlinearity for PIF, and nonlinear transfer function for LIF neurons), we needed to numerically solve a very high-dimensional nonlinear equation. We resorted to the iterative process described in Eq. (9) and Eq. (13), respectively. Although this yielded robustly converging results in all cases without exception, it was not a very fast, sometimes an extremely slow procedure. The spiking network, in contrast, reaches to the solution much faster, i.e. as soon as the onset transient is over and stationary spiking is reached (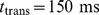 in all our simulations); it is only necessary to record enough spikes to reliably estimate the firing rate, especially if the activity is very sparse. It might therefore be interesting to see if the spiking network dynamics can be exploited to efficiently solve high-dimensional nonlinear equations numerically, especially as the nonlinear solver becomes very slow for high dimensions.

## Methods

### Neuron model

The sub-threshold dynamics of the membrane potential 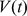 of LIF neurons is described by the leaky integrator equation
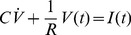
(1)where the resting membrane potential 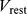 is set to 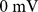. The current 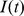 represents the total input to the neuron, the integration of which is governed by the leak resistance 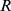, the membrane capacitance 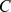, and the resulting membrane time constant 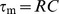. When the voltage reaches the threshold at 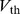, a spike is generated and transmitted to all postsynaptic neurons, and the membrane potential is reset to rest. It remains at this level for a brief absolute refractory period, 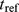, during which all synaptic currents are shunted.

The PIF neuron model lacks the leak term in Eq. 1, which is equivalent to an infinitely large leak resistance, or zero leak conductivity, 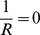. The dynamics of the membrane potential then amounts to
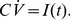
(2)


This is the perfect integrator equation.

In a recurrent network of integrate-and-fire neurons (e.g., PIF or LIF), the total input current 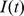 is a linear superposition of all individual post-synaptic currents (PSC). Here, we consider the case where the total charge 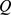 transmitted to the post-synaptic neuron is delivered instantaneously during a pulse-like post-synaptic current. As a consequence, the post-synaptic potential (PSP) induced by each incoming spike has an amplitude of 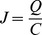. For a LIF neuron, it then decays exponentially with a time constant 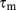. For a PIF neuron, the post-synaptic potential does not decay, but remains at the level reached until another input event changes it.

Note that the absence of a leak term in the PIF model is practically equivalent to having a very large membrane time constant, 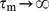, a property that we have exploited in our numerical simulations. The implementation of both LIF and PIF neuron models is based on a numerical method known as “exact integration” [45], [46]. Numerical simulations of all networks were performed using the neuronal simulation environment NEST [47].

### Firing rate model

The net change in the membrane potential of a PIF neuron can be expressed in terms of all incoming spikes. After each output spike, the membrane potential is reset to rest, so we also need to account for the size of this voltage jump 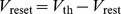, which effectively hyperpolarizes the neuron. If 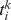 is the time of the *k*-th spike elicited by neuron 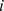, we use a Dirac delta-function 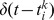 to represent it as a temporal signal. The sum 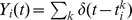 then stands for the spike train of neuron 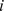. We use 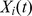 to denote the spike train corresponding to the external stimulus and obtain the following equation to describe the dynamics of a network with synaptic couplings encoded in the connectivity matrix 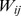


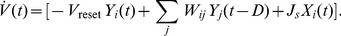
(3)


Assuming stationarity, there can be no drift of the time averaged membrane potential, and we have 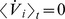. We write 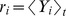 for the mean firing rate of neuron 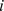 in the network, and 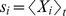 for the mean firing rate of its external input (stimulus), respectively. Under these conditions, we therefore obtain an equation that relates the stationary firing rates of all neurons in the network to their input firing rates
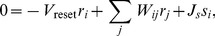
(4)


Observe that transmission delays are irrelevant for temporal averages, and that the above equation holds for networks with arbitrary connectivity.

For a network of 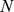 neurons, the recurrent synaptic connectivity is encoded by a fixed 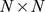 coupling matrix 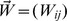. The external inputs and the firing rates of all neurons are represented by the *N*-dimensional vectors 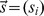 and 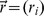, respectively. The time averaged equation above then reads, in matrix-vector notation
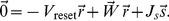
(5)


If all firing rates are non-negative, this equation can be solved for the vector 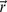 of recurrent firing rates by linear methods
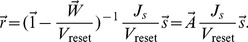
(6)


The only difference in the case of LIF neurons is the additional leak term, which after time averaging leads to a term proportional to the mean membrane potential (see [21], [30], [48]). Eq. 4 should now be written as
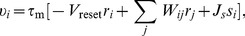
(7)where 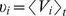 is the time averaged membrane potential of neuron 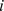. Similarly, instead of Eq. 6, one now obtains
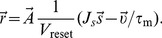
(8)


A PIF network is therefore equivalent to a LIF network with a mean membrane potential clamped at 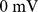.

### Nonlinear firing rate equation

In this section, we extend our linear analysis to account for the nonlinearity of rectification. To obtain the linear rectified tuning curves in Fig. 5B, we artificially applied the rectification on the output of the linear prediction. This is obviously not an exact solution. To improve the result, the effect of silent neurons with rectified firing rates must be considered in the dynamics of network, as their contributions to network dynamics are effectively shunted.

To obtain this, we should solve instead the nonlinear firing rate equation:
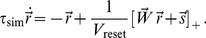
(9)


In order to obtain the population response of the network to each orientation, we solved this equation iteratively for 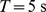 and 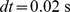, and chose 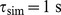.

The nonlinear analysis provided here became possible since a linear rectified response function could be attributed to PIF neurons. How could this strategy be extended to networks of LIF neurons? This is more challenging since the output firing rate of LIF neurons is known to depend on both the mean and the standard deviation of the input, and hence they show a more complicated nonlinear input-output curve. This is in contrast to PIF neurons, which are only sensitive to the mean net input.

In fact, equation Eq. 9 can be written in its general form for any nonlinear input-output transfer function. Let 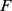 be a nonlinear transfer function which determines the response of an arbitrary neuron model to its dynamic input, as a function of 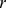 and 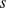. Eq. 9 can now be rewritten as
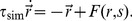
(10)


In case of a network of LIF neurons, this function is explicitly known, given the first two moments of the input, the mean 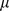 and standard deviation 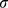


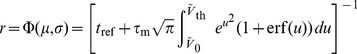
(11)with 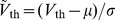 and 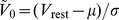. Here 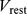, is the resting voltage, to which the neuron is reset after each spike, and 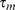 is the membrane time constant.

The mean input can be expressed as 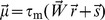. Under the assumption of uncorrelated, Poissonian incoming spike trains, we can write the variance explicitly as
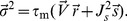
(12)


Here 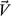 is a matrix obtained by taking the square of each entry in 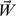, i.e. 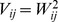. Now, Eq. 9 can be written as
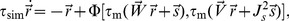
(13)and solved iteratively, as before. To perform this computation more effectively, we have interpolated the firing rates from precomputed combinations of 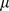 and 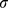.

### Simulation of networks of spiking neurons

We simulate a network of 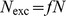 excitatory and 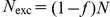 inhibitory neurons. Each neuron in the network receives (1) background input from remote sources, (2) recurrent input from other neurons in the same network, and (3) feedforward sensory input from thalamic neurons in the lateral geniculate nucleus (LGN):

1. The *background input* consists of input from 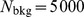 remote neurons, each firing with a spontaneous rate of 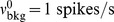, with the synaptic strength of 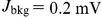. In our simulations, we assume these neurons to fire independently of each other and, therefore, emulate the total input as a homogeneous Poisson process with total rate of 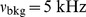.
2. The *recurrent input* is the input that each neuron, be it excitatory or inhibitory, receives from 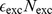 excitatory and 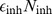 inhibitory neurons randomly sampled from the local network. That is, for all network connectivities, we fix the in-degree, separately for the excitatory and the inhibitory population, with 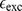 and 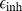 denoting the probability of excitatory and inhibitory connections, respectively. Multiple synaptic contacts and self-contacts are excluded, and the connectivity matrix respects “Dale's rule” [48]. The weight matrix 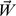 encodes the exact configuration of connections within the local network. The total recurrent input, 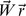, that is received by each neuron depends on the firing rates of all neurons in the network.
3. The *feedforward input* is modeled as a result of the convergence of 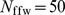 thalamic neurons. The response of each thalamic neuron to the stimulus, 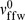, depends on the stimulus contrast, 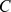, which is a measure of the difference between the stimulus luminance and the background. We take the firing rate to be 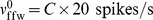, and we simulate the network in response to three different contrasts (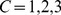). The whole input is again emulated as a Poisson process with total rate 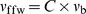, with 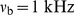. We take the feedforward efficacy to be 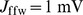.

An oriented stimulus is assumed to induce a weak modulation in the total feedforward input each neuron receives. The modulation is given as a cosine function of the orientation of the stimulus, 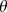, relative to the input preferred orientation (PO) of the neuron, 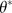. Neuron 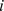 with the input PO of 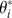 receives a feedforward input of rate 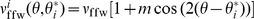. Here, 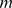 is the input modulation ratio, and determines which fraction of the feedforward input is tuned. The input POs of all neurons are randomly and independently drawn from a uniform distribution on 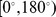. The default parameters of our simulations are summarized in Table 1.

**Table 1:** Table of default parameters.

**Neuron model**		
membrane time constant	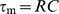	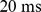 (LIF); 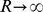 (PIF)
resting potential	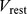	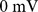
threshold voltage	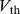	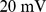
reset voltage	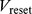	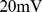
refractory period	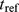	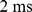
**Synapse model**		
EPSP	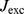	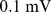
IPSP	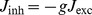	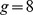 (or 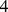 )
feedforward (ffw) EPSP	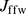	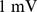
background (bkg) EPSP	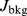	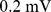
ffw and bkg delays	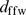 , 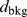	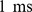
local recurrent delays	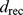	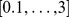 or 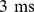
**Network connectivity**		
# neurons	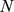	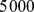
fraction of excitatory neurons	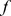	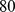 %
fraction of inhibitory neurons	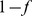	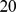 %
# excitatory inputs	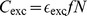	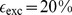
# inhibitory inputs	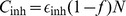	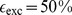
**Simulation**		
stimulus orientation	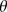	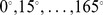
preferred orientation (PO)	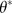	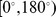
background input rate	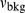	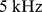
contrast	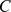	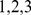
ffw baseline rate	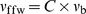	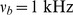
modulation ratio	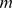	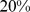

If we denote the vector of input POs by 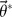, the feedforward input at stimulus orientation 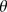 is formed from the components 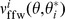. If the background input is added to this input, the output firing rate can be computed from Eq. 6 as:
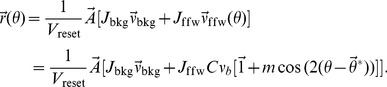
(14)


### Data analysis

To quantify orientation selectivity, we compute the Preferred Orientation, PO, and the Orientation Selectivity Index, OSI, of each neuron from its output tuning curve, 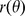, obtained in numerical simulations. We first compute the circular mean [49] of the firing rate measured at each orientation
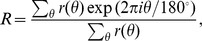
where 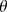 is the stimulus orientation given in degrees. The output PO is extracted as the angle of the resultant, 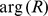, and the length of it, 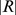, yields a global measure of orientation selectivity, OSI [50].

To each output tuning curve, 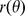, we fit a von Mises function
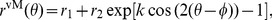
(15)using a nonlinear least squares method. The normalized error of fit is computed from the squared error of the fit
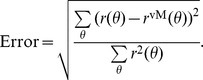



From the best fitting function, the tuning width is extracted as
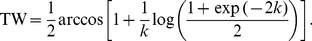
(16)


For each output tuning curve, 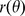, we also compute the baseline (F0) and the modulation (F2) component. The baseline is obtained by averaging the tuning curve over all orientations, and the modulation is obtained from the second Fourier component of the tuning curve.

## Supporting Information

S1 Fig
**Orientation tuning in recurrent networks of either PIF or LIF neurons with broad inhibitory selectivity.** Same as Fig. 4, but inhibitory neurons have broader output tuning curves. All the parameters are the same as before, only the modulation ratio of the input to inhibitory neurons is reduced to 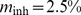. The modulation ratio of the input to excitatory neurons is kept the same as before, i.e. 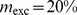. Very similar output excitatory tuning curves are obtained for all three network configurations, only the output tuning curves of inhibitory neurons are broadly tuned now, as a result of receiving a more broadly tuned input. To speed up the simulations, response of each network to each orientation is simulated for 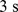.(TIF)

S2 Fig
**Sharpening of output tuning curves in different networks.** (**A–C**) Distribution the output tuning width (TW) is compared among the three networks we considered in Figs. 1–7 at different contrasts. Whereas the PIF network with 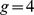 almost preserves the cosine tuning of the input (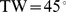) also in its output tuning curves, sharpening is much more prevalent in PIF and LIF networks with 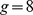, at all contrasts. (**D–E**) The sharpening of tuning curves is quantified by a sharpening coefficient, SC, which is the ratio of the TW of an output tuning curve and the TW of the corresponding input tuning curve: any value less than 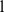 indicates some degree of sharpening. While most of the neurons return a SC close to unity for PIF networks with 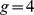 at all contrasts (D), the distribution of SC for PIF and LIF networks with 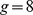 reveals a significant degree of sharpening over the population. The y-axis in all panels is cut at 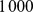 for illustrative purposes.(TIF)

S3 Fig
**Comparison of orientation selectivity in PIF and LIF networks with comparable levels of output firing rates.** (**A–C**) Average output tuning curves of the PIF and LIF networks (same as in Fig. 2 and Fig. 3, respectively) are superimposed for comparison, at different contrasts. The network tuning curves are the same as Fig. 2D and Fig. 3D, and the tuning width (TW) is computed similarly from the best fitted von Mises function. (**D–E**) To compare the tuning curves in networks with a comparable level of output firing rates, the strength of the feedforward input in the LIF network has been increased. That is, all the parameters are kept fixed as in (A–C), only 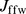 is increased from 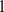 to 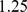, at all contrasts.(TIF)
