# Supplementary figures and images for: Orientation Selectivity in Inhibition-Dominated Networks of Spiking Neurons: Effect of Single Neuron Properties and Network Dynamics
Source: PLoS Comput Biol. 2015 Jan 8;11(1):e1004045. doi: 10.1371/journal.pcbi.1004045 (PMC4287576; doi:10.1371/journal.pcbi.1004045)

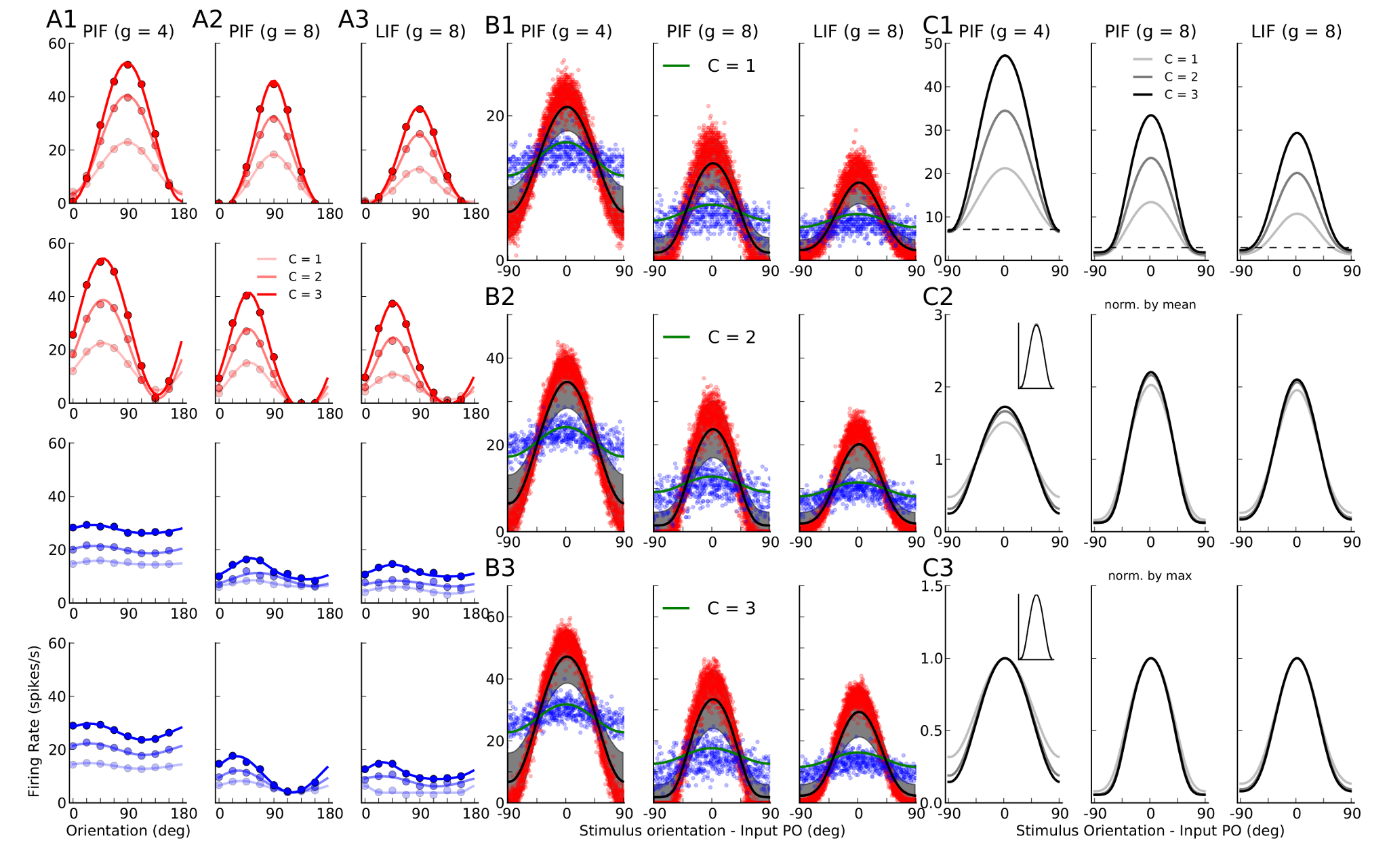

Supplement: S1 Fig — Orientation tuning in recurrent networks of either PIF or LIF neurons with broad inhibitory selectivity. Same as Fig. 4, but inhibitory neurons have broader output tuning curves. All the parameters are the same as before, only the modulation ratio of the input to inhibitory neurons is reduced to . The modulation ratio of the input to excitatory neurons is kept the same as before, i.e. . Very similar output excitatory tuning curves are obtained for all three network configurations, only the output tuning curves of inhibitory neurons are broadly tuned now, as a result of receiving a more broadly tuned input. To speed up the simulations, response of each network to each orientation is simulated for . (TIF) [file pcbi.1004045.s001.tif]

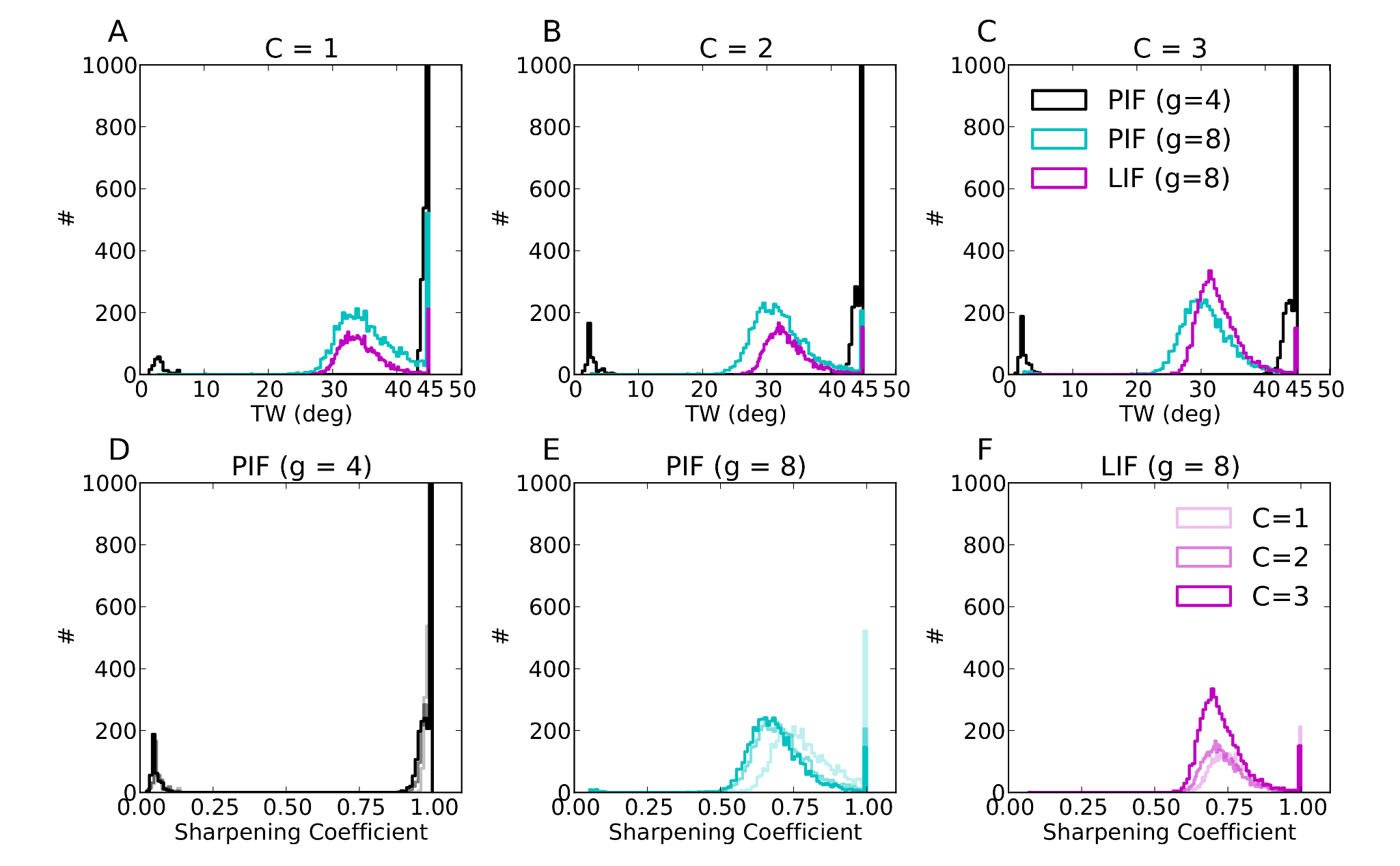

Supplement: S2 Fig — Sharpening of output tuning curves in different networks. (A–C) Distribution the output tuning width (TW) is compared among the three networks we considered in Figs. 1–7 at different contrasts. Whereas the PIF network with almost preserves the cosine tuning of the input () also in its output tuning curves, sharpening is much more prevalent in PIF and LIF networks with , at all contrasts. (D–E) The sharpening of tuning curves is quantified by a sharpening coefficient, SC, which is the ratio of the TW of an output tuning curve and the TW of the corresponding input tuning curve: any value less than indicates some degree of sharpening. While most of the neurons return a SC close to unity for PIF networks with at all contrasts (D), the distribution of SC for PIF and LIF networks with reveals a significant degree of sharpening over the population. The y-axis in all panels is cut at for illustrative purposes. (TIF) [file pcbi.1004045.s002.tif]

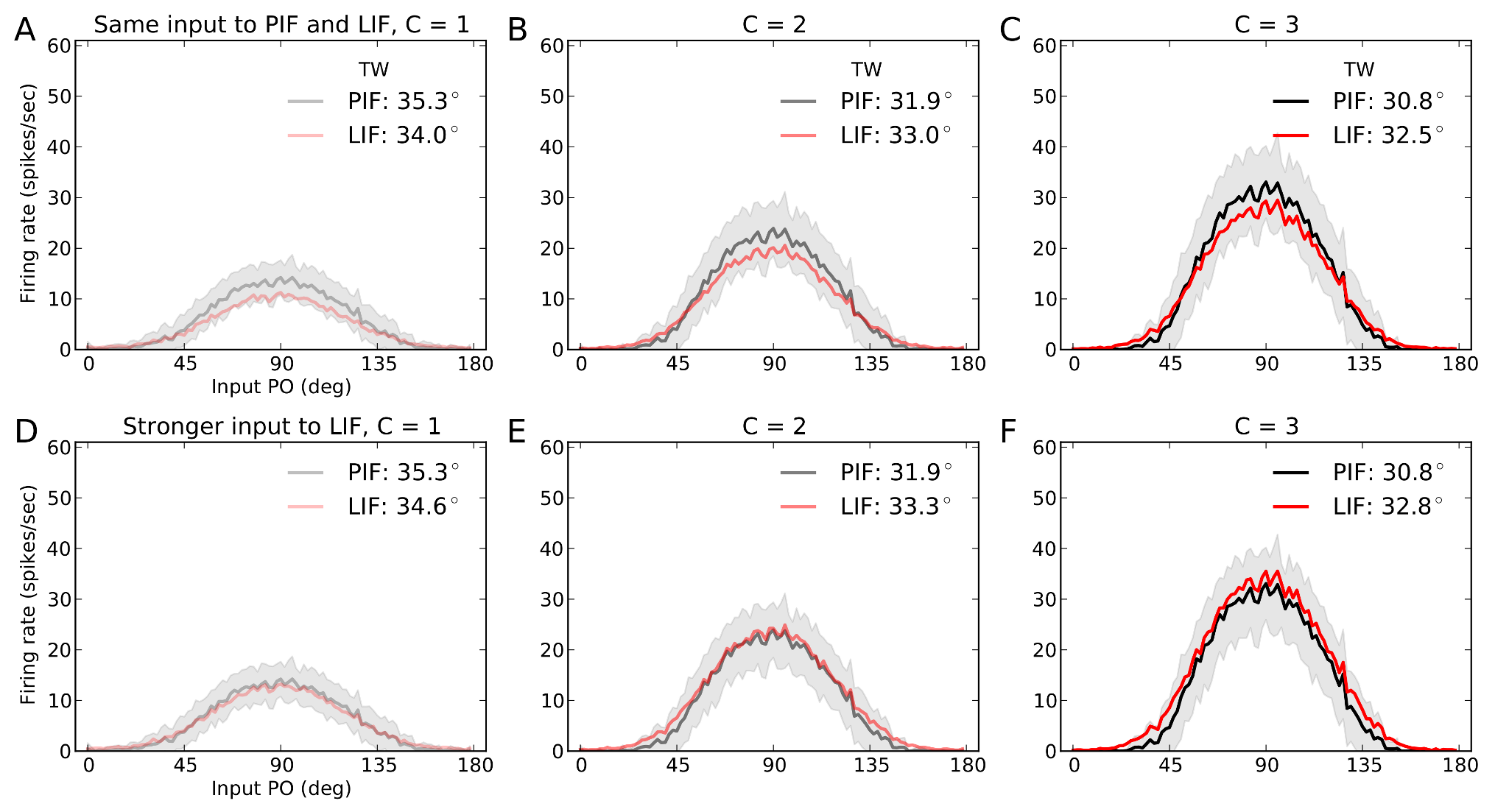

Supplement: S3 Fig — Comparison of orientation selectivity in PIF and LIF networks with comparable levels of output firing rates. (A–C) Average output tuning curves of the PIF and LIF networks (same as in Fig. 2 and Fig. 3, respectively) are superimposed for comparison, at different contrasts. The network tuning curves are the same as Fig. 2D and Fig. 3D, and the tuning width (TW) is computed similarly from the best fitted von Mises function. (D–E) To compare the tuning curves in networks with a comparable level of output firing rates, the strength of the feedforward input in the LIF network has been increased. That is, all the parameters are kept fixed as in (A–C), only is increased from to , at all contrasts. (TIF) [file pcbi.1004045.s003.tif]
